# Circular RNA 0102049 suppresses the progression of osteosarcoma through modulating miR-520g-3p/PLK2 axis

**DOI:** 10.1080/21655979.2021.1923259

**Published:** 2021-06-01

**Authors:** Xianliao Zhang, Zhengbo Hu, Wenhu Li, Zhongxun Liu, Jie Li, Zhaozhen Wang, Vidmi Taolam Martin, Bing Yan, Bo Yu

**Affiliations:** a Department of Orthopedics, Zhujiang Hospital of Southern Medical University, Guangzhou, China; b Department of Orthopedics, Shaoguan First People’s Hospital Affiliated to Southern Medical University, Shaoguan, China

**Keywords:** Osteosarcoma, circ_0102049, miR-520g-3p, PLK2

## Abstract

Circular RNAs (circRNAs) are a type of non-coding RNAs generated from back splicing to enhance or inhibit the progression of multiple human cancers including osteosarcoma (OS). Although circ_0102049 has been found to be highly expressed in OS cell lines, the role and specific mechanism of circ_0102049 in OS remains unclear. Here, we found that silence of circ_0102049 could significantly exacerbate the tumorigenesis of OS *in vivo* through sponging microRNA-520g-3p. Polo-like kinase 2 (PLK2) was predicted to be a target of miR-520g-3p, and luciferase reporter assay revealed that overexpression of miR-520g-3p dramatically suppressed the expression of PLK2, whereas miR-520g-3p inhibitor promoted the PLK2 expression. Moreover, the silence of circ_0102049 could markedly promote the proliferation, invasion, migration and cell-cycle promotion while inhibiting the apoptosis of OS cell line MG63 cells *in vitro* through regulating miR-520g-3p/PLK2 axis. Taken together, the present study indicated that circ_0102049 suppressed the progression of osteosarcoma via modulating miR-520g-3p/PLK2/TAp73 axis, providing a potential therapeutic target for OS.

## Graphical abstract

**Figure:**
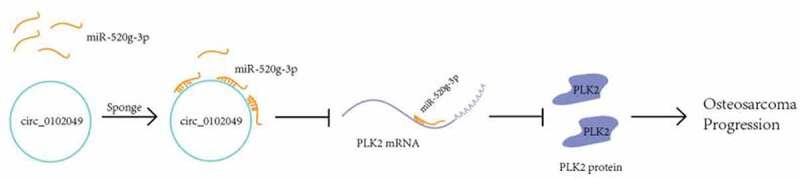

## Introduction

Osteosarcoma (OS), a highly aggressive bone tumor, mainly occurs in children and adolescents and always leads to abnormalities with the long-term survival rates approximately less than 20% [1,2]. In the last decades, the prognosis of patients has dramatically improved due to the introduction of chemotherapy. Owing to the development of multiagent chemotherapy regimens, the long-term survival rates of OS patients have raised to approximately 70%[3]. However, increasing evidence has indicated that some chemotherapy strategies for OS patients induce severe side effects [4,5]. To date, molecular targeted drugs for OS have not been well developed [6], as the molecular events characterizing the occurrence and development of OS have not been well clarified [7]. Hence, it is urgent to explore the underlying mechanisms strongly associated with the progression of OS, which can certainly contribute to identify efficient molecular targets and improve therapies for OS treatment.

Circular RNAs (circRNAs), as a special subclass of non-coding RNAs, have been demonstrated to regulate gene expression by sponging microRNAs in eukaryotes [8,9]. Numerous circRNAs are involved in cancer progression and considered as potential biomarkers for the diagnosis and prognosis of various human tumors [10]. Han et al. demonstrated that circ-BANP enhances the development of lung cancer by modulating miR-503/LARP1 pathway [11]. It has been identified that circ-ITCH suppresses the progression of bladder cancer via directly sponging miR-17 or miR-224 to regulate p21 and PTEN [12]. Yao et al. reported that circ_0058124 enhances the tumorigenesis and invasiveness of papillary thyroid cancer through targeting the NOTCH3/GATAD2A axis [13]. Rong et al. demonstrated that silence of hsa_circ_0007534 suppresses the growth and invasion of cervical cancer cells via the miR-498/BMI-1 pathway [14].

Currently, increasing studies have confirmed the critical roles of circRNAs in the progression of OS. Song et al. reported that circ_0001564 regulates the tumorigenicity of OS as a sponge of miR-29 c-3p, which might be a potential biomarker for OS diagnosis [15]. Xiao et al. revealed that the level of circ_HIPK3 is downregulated in the OS cells, tissues and plasmas of OS patients, which efficiently prohibits the growth, migration and invasion of OS cells [16]. It has been demonstrated that circTADA2A promotes the development and metastasis of OS through directly sponging miR-203a-3p to regulate CREB3 expression [17]. Zhang et al. revealed that circ_001569 is upregulated in OS tumor tissues and enhances growth and cisplatin resistance of OS cells through Wnt/β-catenin axis [18]. In addition, a recent study has indicated that circTP53 contributes to the tumor spectrum and malignant transformation of OS [19]. However, the knowledge of circRNAs in OS is still limited. A previous study has revealed that circ_0102049 is highly expressed in OS cell lines through a high-throughput sequencing technology [20]. However, the roles and specific mechanism of circ_0102049 in OS hhavenot been well studied.

The aim of this study was to investigate the role of circ_0102049 in OS development and identify that whether circ_0102049 suppressed the tumorigenesis of OS through miR-520g-3p/PLK2 axis. Results of this study might provide promising therapeutic targets for OS treatment.

## Materials and methods

### Cell culture

Human osteosarcoma cell-line MG63 cells were obtained from The Cell Bank of Type Culture Collection of Chinese Academy of Sciences (Shanghai, China). MG63 cells were cultured by RPMI-1640 medium supplemented with 10% FBS and 100 U/ml penicillin-streptomycin at 37°C with 5% CO_2_. All cell culture medium and FBS were obtained from Gibco, Invitrogen Corp. (Grand Island, NY, USA).

### Cell transfection

MG63 cells were seeded into 6-well plates at the density of 2 × 10^5^ cells/well and transfected with small interfering RNA (siRNA) (sh-circ_0102049, sh-NC), miRNA mimics (miR-520g-3p mimic, miR-NC), miRNA inhibitors (miR-520g-3p inhibitor, inhibitor NC), or co-transfected with sh-circ_0102049 and miR-520g-3p inhibitor, sh-circ_0102049 and pc-PLK2 (expression vector of PLK2), or miR-520g-3p mimics and pc-PLK2 using Lipofectamine® 3000 kit (Invitrogen). All siRNA, miRNA mimics and inhibitors were synthetized by GenePharma (Shanghai, China). Stable MG63 cells for circ_0102049 silence, miR-520g-3p overexpression or miR-520g-3p silence were established through lentiviral transduction by using the pCDH plasmid (System Biosciences, Mountain View, CA, USA).

### Plasmid construction

For the overexpression of PLK2, the open reading frame of the *PLK2* gene was amplified and cloned into pcDNA3.1 vector. The sequences of *PLK2* gene used in this study were as follows: pc-PLK2 forward: 5ʹ-CGCGGATCCGCCACCATGGAGCTTTTGCGGACTATCACCTAC-3ʹ, reverse: 5ʹ-CCGCTCGAGTCAGTTACATCTTTGTAAGAGCATGTTCAG-3ʹ.

### Luciferase reporter assay

Briefly, the wild type (WT) 3ʹUTR of PLK2 mRNA or mutated 3ʹUTR of PLK2 mRNA (MUT) against the putative binding site of miR-52-g-3p was cloned into the luciferase reporter vector psiCHECK-2 (Promega, Wisconsin, USA). For the luciferase reporter experiments, approximately 1 × 10^4^ cells/well were plated into 24-well plates and treated with the indicated luciferase reporter vectors and miR-520g-3p mimics, miR-NC, miR-520g-3p inhibitor or inhibitor NC by Lipofectamine® 3000 kit (Invitrogen). Next, the relative luciferase activity in cells was detected using the Dual-luciferase Reporter Assay System (Promega) at 48 h after transfection. The primers used for the amplification of WT or MUT 3ʹUTR of PLK2 mRNA were listed as follows: 3ʹUTR WT PLK2 forward: 5ʹ-CCGCTCGAGAAGACTTTTCGAATGGACCCTATGGGAC-3ʹ, reverse: 5ʹ-ATAAGAATGCGGCCGCTTCTGCGTTTTCATACTCTTTATTG-3ʹ; 3ʹUTR MUT PLK2 forward: 5ʹ-AGCATTTCAGCCAGCAACTGGGAGAACTGTGAATATACTTCCTGAAGGGGAGGGAG-3ʹ, reverse: 5ʹ-CACAGTTCTCCCAGTTGCTGGCTGAAATGCTCTCAACAGAGAGAATTTAAGAATCA-3ʹ.

### RNA pulldown assay

RNA pulldown assay was performed with biotinylated WT or MUT miR-520g-3p probes. Briefly, approximately 1 × 10^7^ MG63 cells were seeded into the well of 6-well plates. Next, cells were harvested and sonicated. The biotinylated WT or MUT miR-520g-3p probes were then incubated with streptavidin magnetic beads. Subsequently, the cell lysates were incubated with the biotinylated WT miR-520g-3p probe, biotinylated MUT miR-520g-3p probe or negative control (NC) probe-coated beads overnight at 4°. The bound RNAs were purified by RNeasy Mini Kit (Qiagen, Valencia, CA, USA), and cDNA was reversely transcribed for qRT-PCR analysis with the High-Capacity RNA-to-cDNA™ Kit (Applied Biosystems). The sequences of probes used for RNA pulldown assay were listed as follows: bio-miR-520g-3p WT: 5ʹ-ACAAAGUGCUUCCCUUUAGAGUGU-3ʹ, bio-miR-520g-3p MUT: 5ʹ-AUGGGACACUUCCCUUUAGAGUGU-3ʹ, bio-NC: 5ʹ-UUUGUACUACACAAAAGUACUG-3ʹ.

### CCK-8 (Cell Counting Kit-8) assay

Cell proliferation was detected by a CCK-8 Kit (Beyotime, Shanghai, China). In brief, 1 × 10^3^ MG63 cells were plated into a 96-well plate, and 10 µl CCK-8 solution was added into the well to incubate with cells for 4 h at 37°C. Finally, the absorbance at 450 nm was identified by a Bio-Rad Laboratories microplate reader (Hercules, CA, USA).

### Western blot

The total protein of MG63 cells was extracted by using RIPA lysis buffer (Beyotime) supplemented with protease inhibitor PMSF (Sigma) after transfection. Protein concentration was quantified by BCA kit (Beyotime); then, equal amounts of proteins extracted from cells of each group were separated by 10% SDS-PAGE and subsequently transferred to a Millipore polyvinylidene fluoride (PVDF) membrane (Burlington, MA, USA). After blocking with 5% nonfat milk in Tris-buffered saline (TBS), the PVDF membranes were incubated with primary antibodies including PLK2 (1:1000, ab137539, abcam) and GAPDH (1:2500, ab9485, abcam) at 4°C overnight. GAPDH was used as an internal reference. Next, the membranes were then washed twice by TBS with 0.1% Triton and incubated with HRP-conjugated secondary antibody (1:20000, 4050–05, Southern Biotech) for 2 h at room temperature (RT). Finally, Clarity™ Western ECL substrate (Bio-Rad) was utilized to visualize the protein bands, and quantification analysis was performed by using ImageJ software.

### Analysis of cell apoptosis

Cell apoptosis was evaluated using an Annexin V-FITC/PI apoptosis detection kit (BD Biosciences, San Diego, CA, USA). Briefly, MG63 cells were centrifuged and re-suspended with a binding buffer at 48 h after transfection. Next, the cells were treated with Annexin V-FITC (5 μL) and propidium iodide (PI) (10 μL) in dark for 10 min at RT. Subsequently, the rate of apoptotic cells was evaluated by the flow cytometry (BD Biosciences) and analyzed by the ModFit LT software.

### Analysis of cell‐cycle

In brief, transfected MG63 cells were digested by trypsin and then centrifuged at 300 g for 5 minutes. After washing twice with phosphate buffer saline (PBS), cells were fixed in 70% ethanol at −20°C overnight. Then, cells were incubated with PI (500 uL) in the dark for 10 minutes at RT. Finally, cell cycle was by detected using the flow cytometry (BD Biosciences) and analyzed using FlowJo software.

### Transwell assay

Invasion and migration of MG63 cells were evaluated using the Transwell Chambers (Corning, 8 μm pore) as previously described [21]. Briefly, 1 × 10^5^ MG63 cells were plated into the upper chambers with a FBS-free medium, while a medium containing 10% FBS was filled into the lower chambers. 24 h later, the migrated or invaded cells were fixed by 4% paraformaldehyde (PFA) and stained by crystal violet and counted by a light microscope. The cells that did not migrate or invade into the membrane were scraped using cotton tips.

### Xenograft model

Four-week-old female BALB/c nude mice were purchased from Beijing Vital River Laboratory Animal Technology Co., Ltd. (Beijing, China), and the housing conditions were as follows: normal grade, free to eat and drink. The xenograft mice model of osteosarcoma was constructed as previously described [22]. Briefly, 5 × 10^6^ WT MG63 cells or stable MG63 cells for circ_0102049 silence, miR-520g-3p overexpression or miR-520g-3p silence were subcutaneously inoculated into nude BALB/c mice to generate four groups (n = 6): NC group, sh-circ_0102049 group, miR-520g-3p mimics group, sh-circ_0102049 + miR-520g-3p inhibitor group. Tumor volume was calculated every three days for 34 days by using the following formula: volume = length × width^2^/2. Finally, mice were sacrificed at 34th day after injection, and the size and weight of the tumors were evaluated. Animal experiments in this study were approved by the Animal Ethics Committee of Zhujiang Hospital of Southern Medical University.

### Statistical analysis

All data of this study were presented as the means ± standard deviation (SD), and each experiment was performed three times. SPSS 21.0 software was used for statistical analysis, and the difference between the two groups was identified by Student’s t-test. In addition, P < 0.05 was considered as a significant threshold.

## Results

The aim of this study was to investigate the role of circ_0102049 in OS development and identify that whether circ_0102049 suppressed the tumorigenesis of OS through miR-520g-3p/PLK2 axis. Results of this study might provide promising therapeutic targets for OS treatment. In summary, our study found that circ_0102049 could suppress the progression of OS by activating PLK2 by targeting miR-520g-3e.

### Silence of circ_0102049 promotes the tumorigenesis of OS by targeting miR-520g-3p

**Figure 1.**
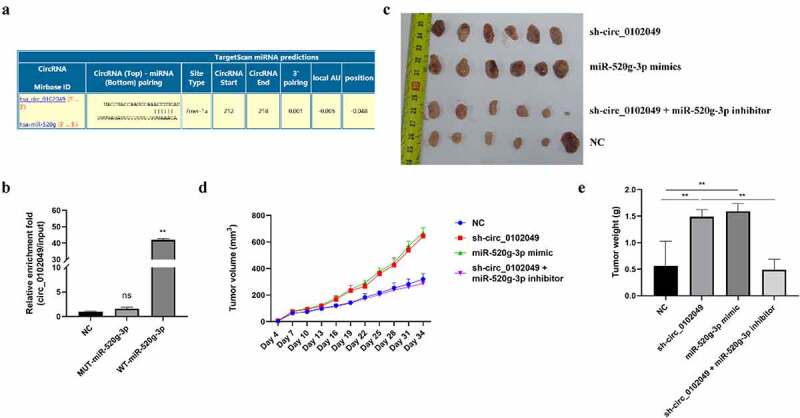
Silence of circ_0102049 promoted the tumorigenesis of OS by sponging miR-520g-3p. (a) The putative binding site between circ_0102049 and miR-520g-3p was predicted by using Targetscan software. (b) The enrichment fold of circ_0102049 was evaluated by RNA pull down assay by using biotin-labeled WT or MUT miR-520g-3p (n = 3). (c-e) Stable MG63 cells which were transfected with sh-circ_0102049, miR-520g-3p mimics, or co-transfected with sh-circ_0102049 and miR-520g-3p inhibitor, were subcutaneously inoculated into nude BALB/c nude mice (n = 6). (c) The representative image of xenograft tumor from different groups harvested at day 34. (d) Tumor volume from different groups was evaluated every three days for 34 days. (e) Tumor weight from different groups was evaluated at day 34. * *P* < 0.05, ** *P* < 0.01 and ns indicates no significant difference

To identify the underlying mechanism of circ_0102049 in OS, Circular RNA Interactome database was used to predict the potential targeted miRNAs of circ_0102049, and results indicated that miR-520g-3p was a potential target of circ_0102049 (Figure 1(a)). Furthermore, RNA pulldown assay was performed by using the biotin-labeled WT or MUT miR-520g-3p probe, and the results indicated that the enrichment fold of circ_0102049 was significantly increased in biotin-labeled WT miR-520g-3p group (p < 0,01), while there was no enrichment of circ_0102049 in biotin-labeled MUT miR-520g-3p group (Figure 1(b)). Above data suggested that circ_0102049 directly interacted with miR-520g-3p. To confirm the role of circ_0102049 and miR-520g-3p in OS, the xenograft mice model was established by using stable MG63 cells for circ_0102049 silence, miR-520g-3p overexpression or miR-520g-3p silence. The xenograft tumors collected at 34th day after transfection are shown in Figure 1(c). Circ_0102049 silence (sh-circ_0102049) and miR-520g-3p overexpression (miR-520g-3p mimics) obviously aggrandized the tumor volume compared with NC group, while co-silence of circ_0102049 and miR-520g-3p (sh-circ_0102049 + miR-520g-3p inhibitor) significantly reversed the effect of circ_0102049 silence (Figure 1(d)). Besides, the tumor weight was significantly increased in circ_0102049 silence (sh-circ_0102049) and miR-520g-3p overexpression (miR-520g-3p mimics) group compared with NC group, while co-silence of circ_0102049 and miR-520g-3p (sh-circ_0102049 + miR-520g-3p inhibitor) dramatically reversed the effect of circ_0102049 silence (Figure 1(e)). These results indicated that silence of circ_0102049 could exacerbate the progression of OS *in vivo* through sponging miR-520g-3p.

### Silence of circ_0102049 upregulates the expression of PLK2 through targeting miR-520g-3p

**Figure 2.**
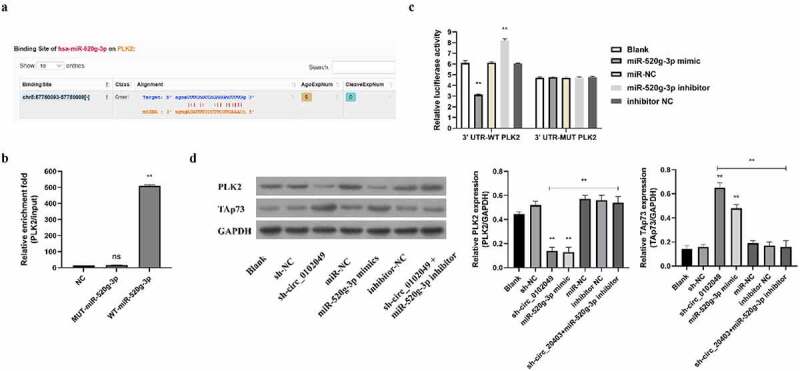
Silence of circ_0102049 suppressed the expression of TAp73 through targeting miR-520g-3p/PLK2 axis. (a) The putative binding site between miR-520g-3p and 3′ UTR of PLK2 was predicted by using Targetscan. (b) The enrichment fold of PLK2 was evaluated by RNA pull down assay by using biotin-labeled WT or MUT miR-520g-3p (n = 3). (c) The luciferase reporter plasmid containing WT or MUT 3′ UTR of PLK2 were co-transfected with miR-520g-3p mimics, miR-520g-3p inhibitor, or corresponding negative control (miR-NC and inhibitor NC) into MG63 cells, and the relative luciferase activity was evaluated by dual luciferase reporter system (n = 3). (d) MG63 cells were transfected with sh-circ_0102049, sh-NC, miR-520g-3p mimics, miR-NC, inhibitor NC, or co-transfected with sh-circ_0102049 and miR-520g-3p inhibitor. 48 h after transfection, the protein expression of PLK2 and TAp73 was evaluated by western blot (n = 3). * P < 0.05, ** *P* < 0.01 and ns indicates no significant difference

MiRNAs often play the crucial roles by inhibiting the expression of their targeted mRNAs as competing endogenous RNAs (ceRNAs) [23,24]. To search for potential targets of miR-520g-3p, Targetscan database was applied and the results showed that PLK2 was a potential target of miR-520g-3p (Figure 2(a)). RNA pulldown assay indicated that the enrichment fold of PLK2 mRNA was dramatically increased in the biotin-labeled WT miR-520g-3p group, while there was no enrichment of PLK2 mRNA in biotin-labeled MUT miR-520g-3p group (Figure 2(b)). Moreover, the results of luciferase reporter assay in MG63 cells transfected with luciferase reporter plasmid contained WT 3ʹUTR of PLK2 mRNA revealed that miR-520g-3p mimics dramatically suppressed PLK2 expression indicated by reduced luciferase activity, whereas miR-520g-3p inhibitor markedly enhanced PLK2 expression indicated by elevated luciferase activity (Figure 2(c)). Besides, both miR-520g-3p mimics and inhibitor had no effect on the miR-520g-3p binding site-mutated PLK2 expression (Figure 2(c)). In addition, we also measured the protein expression of PLK2 in MG63 cells by Western blot. Results indicated that sh-circ_0102049 and miR-520g-3p mimics dramatically suppressed the expression of PLK2, while co-transfection of sh-circ_0102049 and miR-520g-3p inhibitor efficiently abolished the effect of sh-circ_0102049 on the expression of PLK2 (Figure 2(d)). These results demonstrated that sh-circ_0102049 could upregulate the expression of PLK2 through targeting miR-520g-3p.

### Circ_0102049 regulates the proliferation, apoptosis and cell cycle of MG63 cells through modulating miR-520g-3p/PLK2 pathway in vitro

**Figure 3.**
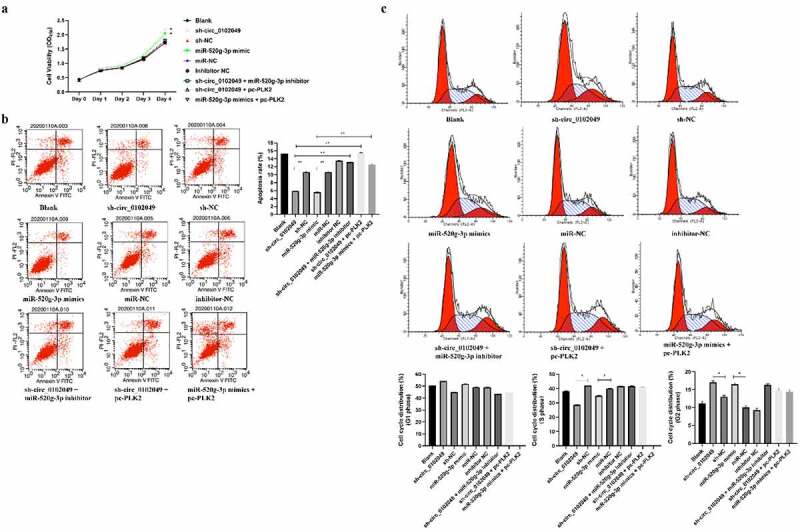
Silence of circ_0102049 promoted the growth, inhibited apoptosis, and promoted G2 phase arrest of MG63 cells through modulating miR-520g-3p/PLK2. MG63 cells were transfected with sh-circ_0102049, sh-NC, miR-520g-3p mimics, miR-NC, inhibitor NC, sh-circ_0102049 + miR-520g-3p inhibitor, sh-circ_0102049 + pc-PLK2 or miR-520g-3p mimics + pc-PLK2. (a) Cell proliferation was evaluated by CCK- assay. (b and c) Cell apoptosis (b) and cell cycle (c) was evaluated by flow cytometry. N = 3, * *P* < 0.05, ** *P* < 0.01 and no marks indicates no significant difference

To explore whether pc-PLK2 (overexpression of PLK2) could reverse the effect of the sh-circ_0102049 and miR-520g-3p mimics on the progression of MG632 cells including growth, apoptosis and cell cycle, MG63 cells were transfected with sh-circ_0102049, sh-NC, miR-520g-3p mimics, miR-NC, sh-circ_0102049 + miR-520g-3p inhibitor, inhibitor NC, sh-circ_0102049 + pc-PLK2 or miR-520g-3p mimics + pc-PLK2. CCK-8 assay showed that sh-circ_0102049 and miR-520g-3p mimics obviously promoted the growth of MG63 cells, while additional miR-520g-3p inhibitor or pc-PLK2 significantly reduced sh-circ_0102049 or miR-520g-3p caused accelerated growth of MG63 cells (Figure 3(a)). Flow cytometry indicated that sh-circ_0102049 and miR-520g-3p mimics significantly reduced the apoptosis of MG63 cells, while additional miR-520g-3p inhibitor or pc-PLK2 obviously reversed the inhibitory effect of sh-circ_0102049 or miR-520g-3p on MG63 cell apoptosis (Figure 3(b)). In addition, the cell cycle was also analyzed and the results indicated that there was no obvious change in G1 phase; sh-circ_0102049 and miR-520g-3p decreased the cell proportion of MG63 cells compared with corresponding controls, while additional miR-520g-3p inhibitor or pc-PLK2 reversed the effect; sh-circ_0102049 and miR-520g-3p dramatically increased the cell proportion of MG63 cells, while additional miR-520g-3p inhibitor or pc-PLK2 reversed the effect (Figure 3(c)), suggesting that sh-circ_0102049 and miR-520g-3p mimics promoted G2 phase arrest of MG63 cells.

### Silence of circ_0102049 enhances the migration and invasion of MG63 cells through regulating miR-520g-3p/PLK2 axis in vitro

**Figure 4.**
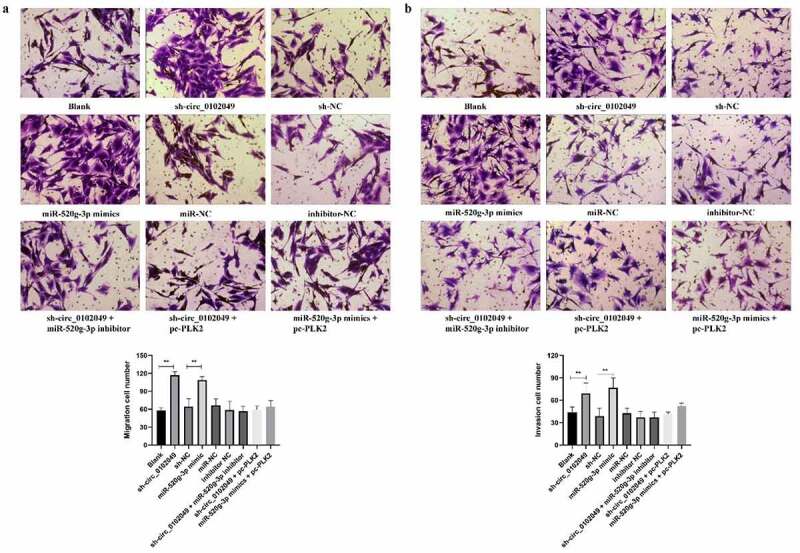
Silence of circ_0102049 promoted the invasion and migration of MG63 cells through targeting miR-520g-3p/PLK2 axis. MG63 cells were transfected with sh-circ_0102049, sh-NC, miR-520g-3p mimics, miR-NC, inhibitor NC, sh-circ_0102049 + miR-520g-3p inhibitor, sh-circ_0102049 + pc-PLK2 or miR-520g-3p mimics + pc-PLK2. The migration (a) and invasion (b) of MG63 cells was evaluated by Transwell assay. Bar: 50 μM, N = 3, ** *P* < 0.01 and no marks indicates no significant difference

**Figure 5.**
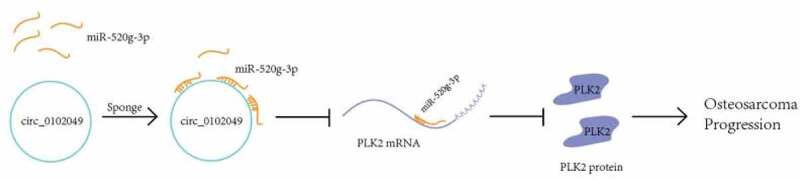
A schematic diagram to summarize the interactions discovered in this study

In addition, Transwell assay indicated that sh-circ_0102049 and miR-520g-3p mimics significantly enhanced migration and invasion of MG63 cells compared to the corresponding control group, while additional miR-520g-3p inhibitor or pc-PLK2 both reversed the effect of sh-circ_0102049 and additional pc-PLK2 reversed the effect of miR-520g-3p mimics (Figure 4(a,b)). In other words, silence of circ_0102049 efficiently enhanced the invasion and migration of MG63 cells through regulating miR-520g-3p/PLK2 axis (Figure 5).

## Discussion

OS, the most common primary bone malignancy, has the highest fatality rate of all cancer-caused death, especially among children and adolescents in the world [25,26]. Therefore, it is necessary to identify and investigate efficient molecular targets for OS treatment. Recently, more and more studies have revealed the crucial roles of circRNAs in carcinogenesis, including the development and metastasis of human tumors, such as colorectal cancer [27], bladder cancer [28], pancreatic cancer [29], lung cancer [30], gastric cancer [31] and so on. For example, circ-DONSON facilitates the proliferation and invasion of gastric cancer cells by activating NURF complex-mediated transcriptional expression of SOX4 [32]. Circ-ZKSCAN1 suppresses the progression of bladder cancer through modifying p21 expression via directly sponging miR-1178-3p, which is considered as a potential prognostic signature of recurrence [33]. In addition, numerous circRNAs are abnormally expressed in various tumors [34].

In OS, a previous study has revealed that circRNA_103801 and circ_0102049 is significantly upregulated, while circRNA_104980 is downregulated in both OS cell lines and tissues through a microarray expression profile analysis [20]. Moreover, there was only one study demonstrated that circ_0102049 functions as a ceRNA of MDM2 to enhance OS progression by directly sponging miR-1304-5p [35]. However, the specific molecular mechanisms of circ_0102049 in OS have not been well understood. In this study, to further explore more mechanisms under which circ_0102049 regulates OS, Circular RNA Interactome database was used to search for the potential targeted miRNAs of circ_0102049 and found that circ_0102049 was predicted to be a potential sponge of miR-520g-3p. RNA pulldown assay confirmed that circ_0102049 interacted with miR-520g-3p. Moreover, the experiments *in vivo* indicated that silence of circ_0102049 (sh-circ_0102049) and overexpression of miR-520g-3p (miR-520g-3p mimics) could significantly promote the growth of xenograft tumors, while the rescue experiments by sh-circ_0102049 + miR-520g-3p inhibitor efficiently reversed the tumorigenic effect of sh-circ_0102049 compared with sh-circ_0102049. These results revealed the tumor-suppressing role of circ_0102049 in OS.

As a tumor inhibitor, the expression of circ_0102049 could be induced to suppress OS during OS development. However, the increase of circ_0102049 expression is not enough to suppress OS development in most cases. Therefore, we guessed that the overall survival of OS patients with high circ_0102049 expression should be longer than those with low circ_0102049 expression.

As we have known, miRNAs always can function as ceRNAs of their target genes to regulate a number of biological processes in eukaryotic cells including malignant tumor [36]. Next, Targetscan was also applied to explore the potential targets of miR-520g-3p, and results indicated that there was a putative binding site of miR-520g-3p in 3ʹ UTR of PLK2 mRNA, suggesting that PLK2 might be a direct target of miR-520g-3p. Both RNA pulldown assay and luciferase reporter assay determined that miR-520g-3p suppressed PLK2 expression through targeting PLK2. Since Llamazares et al. firstly identified PLK in 1991 [37], there are five members of the PLK family including PLK1, PLK2, PLK3, PLK4, and PLK5 have been uncovered [38]. Of which, several studies have revealed that PLK2 plays critical roles in OS cells. For instance, Shen et al. found that the upregulation of PLK2 expression by GATA-1 acetylation can affect the progression of OS in human OS MG63 cells [39]. Here, to explore whether the expression of PLK2 was regulated by circ_0102049 or miR-520g-3p, MG63 cells were transfected with sh-circ_0102049, miR-520g-3p mimics, sh-circ_0102049 plus miR-520g-3p inhibitor, or their corresponding negative control. The results of Western blot assay indicated that sh-circ_0102049 and miR-520g-3p mimics significantly reduced the expression of PLK2, while the rescue experiments by sh-circ_0102049 plus miR-520g-3p inhibitor markedly reversed the inhibitory effect of sh-circ_0102049 on the PLK2 expression. These results suggested that circ_0102049 could affect the PLK2 and expression through sponging miR-520g-3p *in vitro*.

It has been reported that PLK2 is also a member of the serine/threonine protein kinase family, which contributes to cell-cycle regulation in MG63 cells [39,40]. Hence, we also evaluated whether the effect of circ_0102049/miR-520g-3p in the growth, apoptosis and cell cycle of OS cell line MG63 cells mediated by PLK2. As expected, both sh-circ_0102049 and miR-520g-3p mimics could promote the growth and G2 phase arrest and inhibit apoptosis of MG63 cells, while the rescue experiments of overexpression of PLK2 including sh-circ_0102049 + miR-520g-3p inhibitor, sh-circ_0102049 + pc-PLK2, or miR-520g-3p mimics + pc-PLK2 obviously reversed the effect of sh-circ_0102049 and miR-520g-3p mimics in the growth, apoptosis and cell cycle. These data demonstrated that circ_0102049 suppressed the progression of OS by modulating miR-520g-3p/PLK2 axis.

However, whether the overexpression of PLK2 could reverse the effect of sh-circ_0102049 or miR-520g-3p mimics in the OS tumorigenesis *in vivo* needed to be explored in the future studies.

## Conclusion

In summary, our study explored the specific mechanism of circ_0102049 in OS and found that circ_0102049 could suppress the progression of OS by activating PLK2 by targeting miR-520g-3e (Figure 5). Our results suggested that circ_0102049 and miR-520g-3p might be potential therapeutic targets for OS treatment.

## Data Availability

The data of this study are available from the corresponding author upon reasonable request.

## Highlights

1. Silence of circ_0102049 promotes the tumorigenesis of OS by targeting miR-520g-3p.
2. Circ_0102049 regulates OS cell proliferation, migration and invasion through miR-520g-3e/PLK2 axis.
3. Circ_0102049 and miR-520g-3e might be novel therapeutic target of OS.
